# A Scoping Review of the Implications and Applications of Body Composition Assessment in Locally Advanced and Locally Recurrent Rectal Cancer

**DOI:** 10.3390/cancers17050846

**Published:** 2025-02-28

**Authors:** Dinh Van Chi Mai, Ioanna Drami, Edward T. Pring, Laura E. Gould, Jason Rai, Alison Wallace, Nicola Hodges, Elaine M. Burns, John T. Jenkins, on behalf of the BiCyCLE Research Group

**Affiliations:** 1 St Mark’s Hospital and Academic Institute, St Mark’s The National Bowel Hospital, London HA1 3UJ, UK; 2 Department of Surgery and Cancer, Imperial College London, London SW7 2AZ, UK; 3 Department of Digestion, and Reproduction, Imperial College London, London W12 0NN, UK; 4 School of Cancer Sciences, College of Veterinary & Life Sciences, University of Glasgow, Glasgow G12 8QQ, UK

**Keywords:** rectal cancer, pelvic exenteration, neoadjuvant therapy, body composition, sarcopenia, myosteatosis, chemotherapy, radiotherapy

## Abstract

**Background**: A strong body of evidence exists demonstrating deleterious relationships between abnormal body composition (BC) and outcomes in non-complex colorectal cancer. Complex rectal cancer (RC) includes locally advanced and locally recurrent tumours. This scoping review aims to summarise the current evidence examining BC in complex RC. **Methods**: A literature search was performed on Ovid MEDLINE, EMBASE, and Cochrane databases. Original studies examining BC in adult patients with complex RC were included. Two authors undertook screening and full-text reviews. **Results**: Thirty-five studies were included. Muscle quantity was the most commonly studied BC metric, with sarcopenia appearing to predict mortality, recurrence, neoadjuvant therapy outcomes, and postoperative complications. In particular, 10 studies examined relationships between BC and neoadjuvant therapy response, with six showing a significant association with sarcopenia. Only one study examined interventions for improving BC in patients with complex RC, and only one study specifically examined patients undergoing pelvic exenteration. Marked variation was also observed in terms of how BC was quantified, both in terms of anatomical location and how cut-off values were defined. **Conclusions**: Sarcopenia appears to predict mortality and recurrence in complex RC. An opportunity exists for a meta-analysis examining poorer BC and neoadjuvant therapy outcomes. There is a paucity of studies examining interventions for poor BC. Further research examining BC specifically in patients undergoing pelvic exenteration surgery is also lacking. Pitfalls identified include variances in how BC is measured on computed tomography and whether external cut-off values for muscle and adipose tissue are appropriate for a particular study population.

## Simple Summary

Body composition refers to the quantity and quality of muscle and different types of fat within a person. In non-advanced cancer affecting the colon and rectum, there is strong evidence that abnormal body composition, such as low muscle quantity (sarcopenia) and lower muscle quality (myosteatosis), can predict poorer outcomes. This review aims to summarise the current research assessing body composition in rectal cancers deemed to be advanced. Across the 35 studies included, the theme observed was that patients with sarcopenia both passed away sooner and had an increased likelihood of the cancer coming back. Furthermore, in some studies, body composition appeared to predict how effective radiotherapy was at shrinking advanced rectal cancers and how well patients tolerated side effects. However, limitations noted across the studies included large differences in how low muscle quantity was defined and how it was measured.

## 1. Introduction

Body composition (BC) quantifies an individual’s proportions of skeletal muscle (SM), adipose tissue (AT), bone, and organs [1]. Point-of-care cross-sectional imaging such as computed tomography (CT) and magnetic resonance imaging (MRI) allow for direct and opportunistic BC measurement, with surface areas of SM and AT from a single axial MRI or CT lumbar slice highly correlating with whole BC [2,3]. Subsequently, substantial evidence has emerged, independently linking phenotypes such as sarcopenia (reduced muscle mass and function), myosteatosis (adipose tissue infiltration of muscle), and adiposity to survival in solid and haematological cancers [4,5,6,7,8].

Colorectal cancer accounts for approximately 10% of global cancer incidence and death, and earlier-onset disease is increasing [9,10]. A comprehensive meta-analysis by Trejo-Avila et al. (2021) showed sarcopenia to independently predict mortality, recurrence, and postoperative complications in colorectal cancer [11]. Rectal cancer (RC) is clinically, molecularly, and biologically distinct compared to colonic cancer [12]. Non-complex primary RC and stage I-III colon cancers are both typically treated with mesorectal/mesocolic excision followed by adjuvant chemotherapy for high-risk histological features. However, multi-modal treatment options deviate for *complex RC*, describing locally advanced RC (LARC) or locally recurrent RC (LRRC). LARC may initially be treated with neoadjuvant chemoradiotherapy (nCRT) [13]. nCRT can not only downsize tumours but may also elicit a complete response (CR). Total neoadjuvant therapy (TNT), where full systemic chemotherapy is administered either as induction or as consolidation with nCRT, can double the CR rates to between 20 and 38% in trials [14]. Even more recently, anti-Programmed Death 1 Ligand 1 (PD-L1) immunotherapy for microsatellite instability-high or mismatch repair-deficient tumours potentially reach 75–100% CR [15,16]. Surgical treatment for complex RC also distinctly differs from colonic cancer. Pelvic exenteration surgery is now an established potentially curative option for LARC, extending beyond the TME plane despite neoadjuvant therapy, as well as for LRRC [17]. However, increased operative times, blood loss, and a markedly greater extent of dissection results in higher surgical stress than contemporary total mesorectal excision (TME), reflected in overall and major complication rates of 71.7% and 23.9%, respectively [18].

Given the distinct disease and treatment features of complex RC, assessing this group would be valuable in determining whether BC predicts postoperative complications, long-term oncological outcomes, and neoadjuvant therapy response and tolerance. Conversely, understanding iatrogenic chemoradiation or exenteration-induced BC changes may help guide prehabilitation. This scoping review aims to summarise the current literature examining BC within complex RC in terms of geographical origin, study designs, patient populations, treatment, methods of BC measurement, outcome measures, and findings. By mapping the literature, opportunities for a more targeted systematic review and meta-analysis can be identified. This will highlight knowledge gaps for future research, as well as potential pitfalls observed in the current literature.

## 2. Materials and Methods

A scoping review was performed in accordance with the five-step process recommended by Arksey et al. (2005) [19] and principles of the Preferred Reporting Items for Systematic Reviews and Meta-Analyses (PRISMA) statement for scoping reviews [20].

### 2.1. Search Strategy

A literature search was performed on Ovid MEDLINE, EMBASE, Cochrane Central Register of Controlled Trials, and Cochrane databases. Headings and keywords included combinations of rect*, cancer, neoplasm, carcinoma, exenteration, sacrum, sacral, sacrectom*, advances, recurrent, recurrence, complex, beyond TME, body composition, body fat distribution, adiposity, visceral, sarcop*, muscl*, myosteatosis, total psoas area, hypoalbuminemia, nutritional status, malnutrition, cachexia, cardio, cardiopulmonary exercise test, walk test, exercise tolerance, physical fitness, and cardiorespiratory fitness.

### 2.2. Definitions

No internationally agreed definitions for LARC exist in research and clinical practice. For this review, a range of definitions for LARC were permitted as follows: (1) T3 or T4-stage disease; (2) tumour or tumour deposits threatening or involving the circumferential resection margin; (3) any lymph node involvement; (4) the presence of extramural vascular invasion; and (5) tumour invasion or adherence to local structures [21,22]. LRRC was defined as the recurrence of RC within the pelvis after previous surgical resection [23]. Complex RC will be an umbrella term for both LARC and LRRC.

### 2.3. Inclusion Criteria

Original studies fulfilling the following were included: (1) adults older than 18 years with complex RC and (2) either examined BC as an independent or dependent variable. Restrictions were not placed upon the method of BC measurement, BC metrics measured, or study outcomes.

### 2.4. Exclusion Criteria

The following were excluded: (1) non-original studies; (2) publications unavailable in English; (3) conference abstracts; (4) studies examining non-gastrointestinal cancers; and (5) studies involving non-complex and complex RC without a subgroup analysis of the latter.

### 2.5. Study Selection

The online *Covidence* systematic review tool (https://www.covidence.org/) was used in this scoping review [24]. Two authors undertook abstract screening and full-text reviews. A senior author was designated to resolve inclusion disagreements. Whilst systematic reviews and meta-analyses were excluded, referenced studies missing from the initial search that fulfilled inclusion criteria also underwent a full-text review.

### 2.6. Data Extraction

Data extraction was performed by a single author using an a priori proforma, summarised in Table S1.

### 2.7. Statistical Analyses

Descriptive statistics summarised study characteristics as frequency counts, as well as medians and interquartile ranges (IQRs) where appropriate. Due to the design of a scoping review, neither formal study quality assessment nor quantitative data synthesis were performed.

## 3. Results

### 3.1. Search Process

**Figure 1:**
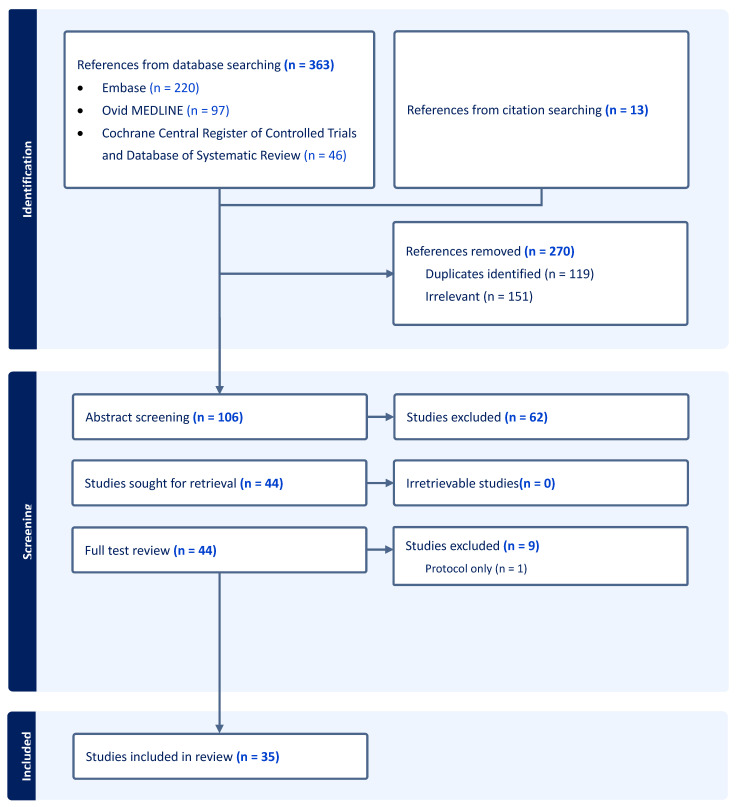
PRISMA flowchart for identification, exclusion, and selection of studies for scoping review.

The PRISMA flowchart for study identification and selection is summarised in Figure 1. In total, 376 articles were initially identified, including 12 from the citation checking of relevant systematic reviews. After the exclusion of irrelevant and duplicate articles, 106 studies underwent abstract screening. A full-text review was performed for 44 studies, from which a further 9 were excluded. The final 35 included studies underwent data extraction, totalling 5669 patients [25,26,27,28,29,30,31,32,33,34,35,36,37,38,39,40,41,42,43,44,45,46,47,48,49,50,51,52,53,54,55,56,57,58,59].

### 3.2. Study Characteristics

**Figure 2:**
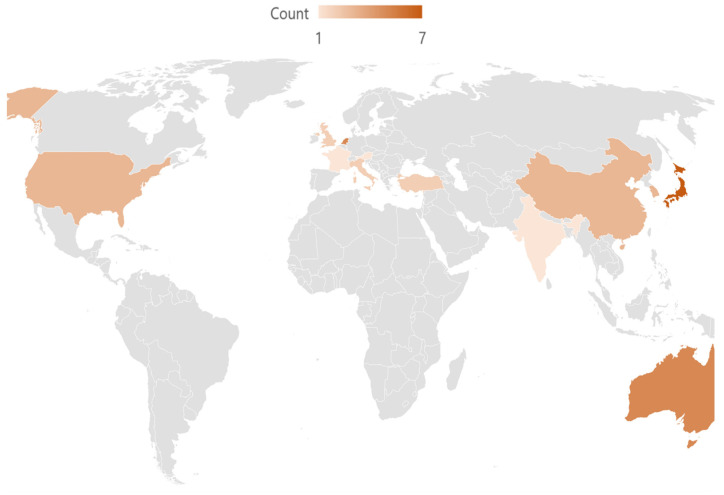
World heat map of study origin.

Table S1 provides a detailed record of the individual characteristics of each study; Table 1 summarises these overall. Of the 35 studies, 24 (68.6%) originated from East Asia and Europe [26,27,28,29,30,31,32,34,35,36,38,39,40,41,43,44,45,46,49,51,53,54,57,58]; the remaining 11 (31.4%) came from Australia, North America, and West and South Asia [25,33,37,42,47,48,50,52,55,56,59] (Figure 2). The median (IQR) sample size was 99 (61–214) patients.

### 3.3. Study Aims

Thirty-one (88.6%) studies aimed to investigate relationships between BC and complex RC outcomes [25,27,28,29,30,31,32,33,34,35,36,37,38,40,41,42,43,44,45,46,48,49,50,51,52,53,54,55,56,57,58]; three generated BC-based predictive models [40,53,54]. Eight (22.9%) examined the effect of nCRT on BC [26,27,32,34,36,39,41,56]. Melucci et al. (2022) compared BC from CT versus MRI [47], whilst Yadav et al. (2024) examined whether prehabilitation could improve BC during nCRT [59].

### 3.4. Study Design and Setting

There were three non-randomised experimental studies [26,47,59]; the remaining 32 (94.3%) were cohort studies, of which 31 were either retrospective or retrospective analyses of prospectively collected data. Three studies were multi-centre [36,48,52].

### 3.5. Study Population

All studies included LARC. A geographical variance in the definition of LARC was observed, where six of the eleven (54.5%) European studies included threatened/involved circumferential resection margin (CRM) as a criteria [26,27,30,39,41,46], whilst 11 of the 13 (84.6%) East Asian studies defined it as T3/T4 and/or >N0 disease without specifying CRM involvement [28,29,31,34,36,40,43,44,53,54,58]. Eleven studies did not specify a definition [32,37,38,42,45,47,48,52,55,56,59]. Only two also included LRRC; subgroup analyses were not conducted [38,41].

### 3.6. Treatment Characteristics

All studies investigated patients undergoing nCRT, whilst two also examined TNT [46,48]. Four did not specify operative details for their cohort, since it was not relevant to the study aims and designs [26,38,47,53]. Thirty-one (88.6%) investigated patients undergoing TME [25,27,28,29,30,31,32,33,34,35,36,37,39,40,41,42,43,44,45,46,48,49,50,51,52,54,55,56,57,58,59]; three of these also included pelvic exenteration surgery [27,48,52]. One study (2.9%) solely looked at pelvic exenteration for LARC and LRRC [41].

### 3.7. Outcome Measures

Short-term postoperative outcomes were examined in 17 (48.6%) studies [25,28,29,30,32,33,34,35,37,41,43,45,46,49,51,56,59], whilst overall survival (OS) and/or disease-free survival (DFS) were examined by 20 of 35 (57.1%) studies [25,27,28,29,30,31,32,34,35,36,40,41,43,44,45,49,50,51,57,58]. Ten (28.6%) assessed neoadjuvant therapy response [25,37,42,45,48,52,54,55,56,57] and six (17.1%) examined complications in neoadjuvant/adjuvant treatment [36,38,48,53,57,58].

### 3.8. Body Composition Analysis

CT was used in 32 studies (91.4%) [25,27,28,29,30,31,32,33,34,35,37,38,39,40,41,42,43,44,45,46,47,48,49,50,51,52,53,54,55,56,57,58]. Twenty-seven of these (84.4%) measured BC at the L3 vetebrae [27,28,29,30,31,32,33,35,37,38,39,40,41,43,44,45,46,47,48,49,50,52,53,54,55,57,58]; the remainder used L4 [25,56,59], the umbilicus [34,51], or the mesorectum [42]. L4 [59], L5 [47], or the thigh [26] were used in three (8.6%) MRI-based studies. SM quantity was assessed in 32 (91.4%) studies [27,28,29,30,31,32,33,34,35,37,38,39,40,41,42,43,44,45,46,47,48,49,50,51,52,53,54,55,56,57,58,59]; 11 measured psoas alone [30,34,37,40,43,44,48,52,56,58,59]. There were 12 studies (34.3%) examining AT quantity [25,28,32,35,42,45,46,49,51,54,55,57]. SM radiodensity was examined in six studies [30,41,45,55]. Marked variation existed regarding how SM and AT were quantified utilising CT or MRI, with 14 different software tools identified (Table S1). Furthermore, two of the studies examining psoas muscle [52] estimated surface area based on diameter rather than direct measurement of the whole area (Table S1).

### 3.9. Body Composition Categorisation

There was variation in defining abnormal BC (Table 2). Twelve studies (34.3%) applied median-, tertile-, quartile-, or standard deviation-based cut-offs to measurements [27,29,30,40,41,43,44,45,50,51,54,55]. Eighteen studies (51.4%) applied cut-offs from previous publications [25,27,28,31,32,33,35,37,38,39,41,46,48,49,51,52,57,58,59]. Three papers experimented with both the literature-based cut-offs and tertiles, quartiles, or standard deviations [27,41,51].

The most frequently used L3-based sarcopenia cut-offs were by Prado et al. (2008), Martin et al. (2013), and Caan et al. (2017), derived from North American gastrointestinal and lung cancer cohorts [60,61,62]. These were used in 13 of the 27 studies (48.1%) examining SM [27,28,32,33,35,37,38,41,46,48,49,52,57], including three conducted on Asian populations [28,32,37]. Martin et al. (2013)’s Hounsfield unit cut-offs were also applied to two of six studies examining SM radiodensity [38,41]. Two of the eleven studies examining the psoas muscle alone used psoas-specific cut-off values [63,64] generated from ethnicities matching their study populations [58,59]. Two studies [48,52] utilised L3 psoas-only area measurements but applied the Prado et al. (2008) cut-offs based on all SM within the L3 slice [60].

Eight of twelve studies examining AT [25,28,32,35,46,49,51,57] used cut-offs based on Fujioka et al.’s (1987) umbilical VAT:SAT ratio or the Japan Society for the Study of Obesity (2002) and Doyle et al.’s (2013) respective L3 and umbilical VAT surface areas [65,66,67]. Three of fifteen longitudinal studies [35,39,49] applied Mourtzakis et al.’s (2008) 2% measurement error cut-off when measuring L3-SM, and Miyamoto et al. (2014), who showed >5% L3-SMI loss, predicted reduced overall and progression-free survival in CRC patients undergoing palliative chemotherapy [3,68].

Two studies computed bespoke optimal cut-off values. Fukuoaka et al. (2019) used receiver operator curves to determine cut-off values for psoas muscle percentage decrease after nCRT [34]. Chiloiro et al. (2024) similarly calculated values for SM radiodensity and L3-SAT and VAT quantity [57].

### 3.10. Study Findings

#### 3.10.1. Overall Survival

Amongst 16 studies examining OS (Table 3), ten (62.5%) showed an association between BC and OS [28,29,30,31,32,40,41,44,50,51,57]; eight demonstrated this in multivariate analysis [28,29,30,31,32,44,50,57]. Seven studies demonstrated this with sarcopenia [28,29,30,31,32,44,50], whilst Chilhoiro et al. (2024) found SM radiodensity-defined myosteatosis predicted mortality rather than sarcopenia [57].

#### 3.10.2. Disease-Free Survival

BC predicted DFS in 15 of 18 studies (83.3%) examining recurrence [25,27,28,29,31,34,35,40,43,44,45,49,50,51,57] (Table 3); this was in a multivariate analysis in nine papers [25,27,29,34,43,44,45,49,57]. Clark et al. (2013) and Chiloiro et al. (2024) found that an elevated VAT:SAT ratio or elevated L3 VAT quantity, respectively, predicted a reduced DFS [25,57]; sarcopenia was the independent predictor in the other seven studies [27,29,34,43,44,45,49].

#### 3.10.3. Early Postoperative Outcomes

As shown in Table 3, 10 of 17 studies (58.8%) assessing early postoperative outcomes found associations with BC [30,33,34,35,45,46,49,51,56,59]. Sarcopenia [33,49,51,59], sarcopenic obesity [30,46,51], and an elevated VAT:SAT ratio [46] were associated with overall complications. De Nardi et al. (2019) and Fukuoka et al. (2019) showed sarcopenia specifically predicted severe morbidity [34,35]. In terms of specific complications, Liu et al. (2022) showed reduced SM radiodensity and total AT depletion predicted ileus [45]; anastomotic leakage was associated with sarcopenia in Yassaie et al. (2024) [56], whilst Mallet-Boutboul et al. (2023) found that viscerally obese patients had higher unplanned readmission rates [49]. An increased LOS was associated with visceral and sarcopenic obesity in Bocca et al.’s (2022) and Mallet-Boutboul et al.’s (2023) works [46,49]. All early postoperative outcome associations were shown in univariate analysis only, with the exception of Bocca et al.’s (2022) study, where an elevated V/S ratio was identified as an independent predictor of >Clavien–Dindo Grade I complications [46].

#### 3.10.4. Outcomes of Neoadjuvant Therapy

Nine of the ten studies (90.0%) examining responses found significant associations between sarcopenia [37,48,54,55,56,57], an elevated VAT:SAT ratio [25], SAT depletion [45], and mesorectal AT volume [42] with overall tumour response (partial to complete) to neoadjuvant therapy. Five studies demonstrated this in multivariate analysis [48,54,55,56,57].

Two of six studies (33.3%) examining adverse outcomes of neoadjuvant or adjuvant therapy found BC to be predictive of tolerance. Bedrikovetski(a) et al. (2023) showed that sarcopenic patients had a significantly lower compliance with neoadjuvant radiotherapy in terms of early discontinuation and dose tolerance in univariate analysis [48]. Chiloiro et al. (2024) found sarcopenia to be an independent predictor of neoadjuvant radiotherapy interruption [57].

#### 3.10.5. BC Changes During nCRT

Eleven studies compared BC before and after nCRT. Six found a significant decrease in SM quantity [34,39,40,54,59] or SM mitochondrial function [26]. Of the 10 studies comparing BC changes to outcomes [27,32,34,35,41,45,49,54,56,59], a significant relationship was reported in seven. SM loss during nCRT was associated with mortality [32,34], recurrence [27,34,35,56], reduced tumour response [56], and postoperative complications [34,56,59]; SAT loss was also associated with reduced DFS [35], reduced downstaging, and postoperative complications [45].

#### 3.10.6. Experimental Studies

There were three non-randomised experimental studies. West et al. (2014) performed an in vivo analysis of SM mitochondrial function before and after nCRT using phosphorus MRI spectroscopy of the quadriceps. nCRT was associated with a decline in cardiorespiratory fitness and SM mitochondrial function [26]. Melucci et al. (2022) demonstrated in 16 LARC patients that lean body mass estimates from bioelectrical impedance analysis correlated more strongly with the MRI pelvis L5 slice SM surface area than with the CT L3 slice [47]. Yadav et al. (2024) was the only interventional study. Forty-four LARC patients treated with multimodal prehabilitation (exercise programme; nutritional, alcohol, smoking, and psychological counselling) experienced a significant 1.3% increase in psoas muscle; the control group experienced a 2.9% decrease. There was a 13.7% decrease in sarcopenia status in the treatment arm versus a 12.5% increase in the control group [59].

## 4. Discussion

### 4.1. Current Knowledge and Clinical Applications

The majority of BC research in complex RC has focused on SM and has been conducted using preoperative CT in European and East Asian cohorts undergoing nCRT and TME for LARC. Since this is a scoping review, formal meta-analysis is beyond the scope of this work. However, several trends are observed between BC and complex RC.

#### 4.1.1. Sarcopenia

Muscle quantity was the most ubiquitous metric across the 35 studies; its depletion appears to predict mortality, recurrence, neoadjuvant therapy outcomes, and postoperative complications.

##### Survival

Sarcopenia has primary and secondary aetiologies; the former is part of ageing and frailty, whilst the latter is driven by systemic illnesses such as malignancy [69]. It independently predicts mortality in elderly individuals, with malignancy, cardiovascular, respiratory, liver, and lung disease in a meta-analysis approaching 10,000 patients [70]. This may also apply to complex RC according to the studies identified for this scoping review. Seven studies show independent impact upon OS; nine papers identified similar relationships with DFS. To our knowledge, two meta-analyses have been published examining sarcopenia and prognosis in LARC. Hatt et al. (2023) included five papers (*n =* 598) from this review [27,28,29,32,35], concluding that pretreatment sarcopenia was significantly predictive of poorer OS [HR 1.69 (95% CI 1.15–2.48)] but not DFS [1.07 (95% CI 0.63–1.82)] [71]. Su et al. (2024) performed a similar meta-analysis for RC in general, including a subgroup analysis of patients undergoing nCRT for LARC from four [28,32,40,50] studies in our review, concluding that preoperative sarcopenia predicted both OS [HR 2.44 (95% CI 1.54–3.87)] and DFS [HR 2.16 (95% CI 1.42–3.31)] [72].

##### Neoadjuvant Therapy Response and Tolerance

BC independently predicted responses to neoadjuvant treatment in five [25,37,42,48,57] of ten papers. Sarcopenia predicted pathological complete response (CR) after nCRT in Yang et al.’s (2023) and Wei et al.’s (2024) works and clinical or pathological CR in Yassaie’s work in 2024 [54,55,56]. Bedrikovetski et al. (2023a) showed that sarcopenia predicted clinical or pathological CR specifically after TNT, whilst it was associated with decreased local control by Chiloiro et al. (2024) [48,57]. Non-RC studies examining BC relative to nCRT response is scarce. Neither Grossberg et al. (2016) nor Pai et al. (2018) found BC to be associated with locoregional control after nCRT for head and neck cancers [73,74].

Mechanistic studies are lacking to fully explain a relationship between pre-treatment sarcopenia and tumour response to neoadjuvant radiotherapy and chemotherapy. One explanation could be that sarcopenia observed in non-responsive patients is secondary to a more aggressive tumour phenotype. Tumour size, T4 and/or N2 disease, and mucinous adenocarcinoma are predictors of reduced nCRT response [75,76]. Sarcopenia may be a systemic manifestation of such adverse tumour features arising from cytokine-mediated cancer cachexia.

Another explanation is that sarcopenic patients are more likely to experience dose reduction or early termination of radiotherapy and/or chemotherapy. Sarcopenic patients had a reduced median radiotherapy dose and compliance in Bedrikovetski et al.’s (2023a) study and increased odds of treatment interruption in Chiloiro et al.’s work [48,57]. The body of evidence for sarcopenia and chemotherapy-induced dose-limiting toxicity has been summarised via a meta-analysis by Surov et al. (2021) and via a systematic review by Drami et al. (2021); it is postulated that with the standard approach of chemotherapy dosing using body surface area [BSA], sarcopenic patients have a lower volume of distribution and hence a higher drug concentration [77,78]. In this review, four studies examined BC in relation to chemotherapy toxicity. Van Rees et al. (2021a) and Yang et al. (2024) examined ≥Grade III gastrointestinal complications of capecitabine-nCRT [38,53]; Bedrikovetski et al. (2023a) examined TNT [48]; and Abe et al. (2024) examined dose-limiting complications from adjuvant chemotherapy [58]. Interestingly, no associations between chemotoxicity and sarcopenia were observed.

##### Postoperative Complications

Eight studies showed that sarcopenia predicted overall and major complications after TME, in line with Trejo-Avila et al.’s work (2021) [11]. Primary sarcopenia due to ageing and sarcopenia secondary to malignancy is associated with a pro-inflammatory state. Malietzis et al. (2016) observed significantly elevated preoperative neutrophil/lymphocyte ratios and hypoalbuminaemia in sarcopenic colorectal cancer patients [79]. Furthermore, sarcopenia reflects preoperative malnutrition [80]. A pro-inflammatory and/or malnourished state may lead to impaired immune responses and tissue healing; combined with the burdens of neoadjuvant and surgical treatments, a greater risk of complications would likely occur [79].

#### 4.1.2. Myosteatosis

Myosteatosis, the infiltration of SM by ectopic AT, happens at the intermuscular, intramuscular, and intramyocellular level; reduced SM radiodensity is a convenient surrogate for the latter [81]. It is another unfavourable BC phenotype. Like sarcopenia, it is associated with ageing and is also linked to metabolic diseases, malignancy, and inactivity [82,83]. Broadly, this results in impaired physical function due to reduced force production, fibrosis, impaired capillarisation, and atrophy of SM. Furthermore, myosteatosis places the patient in a pro-inflammatory state, similar to sarcopenia, due to elevations of circulating pro-inflammatory adipokines and myokines [84], as is observed in colorectal cancer patients [79]. Chang et al.’s (2024) meta-analysis showed that myosteatosis independently predicted reduced postoperative OS [HR 1.52 (1.38–1.67)] and DFS [HR 1.89 (1.35–2.65)] in colorectal cancer; MacCormick et al. (2022) similarly showed this across GI cancers in general [85,86]. Only four studies examined SM radiodensity [30,41,45,55]. In LARC treated with nCRT and TME, Berkel et al. demonstrated that myosteatosis was independently associated with overall and major postoperative complications but not OS [30]. Van Rees et al. (2021b) showed that myosteatosis was significantly associated with OS in a univariate analysis but not in multivariate testing [41]. Myosteatosis was associated with postoperative ileus and independently predicted cancer-specific survival in Liu et al. (2022) [45].

#### 4.1.3. Adipose Tissue

Although less useful as a surrogate for adiposity at normal and lower values [87], body mass index (BMI) is moderately to strongly correlated with fat mass [88,89,90]. Obesity is independently associated with greater colorectal cancer risk [91]. However, relationships to outcomes are less obvious. A meta-analysis of 56 studies for colorectal cancer concluded BMI extremities (BMI < 18.5 or BMI > 35) were associated with reduced OS and DFS, whilst being overweight (BMI 25 to 30) conferred a survival advantage [92]. This “obesity paradox” was further investigated by Caan et al. (2017) on 4465 patients by quantified AT via L3 slice analysis. The overweight patients (BMI 25 to 30) had both the longest OS and most optimal BC, i.e., non-sarcopenic with lower abdominal AT quantity [62]. However, a subsequent meta-analysis by Caan’s group concluded that there was no overall relationship between total, subcutaneous, or visceral adiposity and colorectal cancer mortality or recurrence, although marked heterogeneity was recorded [93].

It is perhaps reductive to summarise patients as binary categories of SM and AT; in reality, individuals fall into various permutations of muscle and fat quantity and quality. Sarcopenic obesity, or sarcopenic adiposity, describes individuals simultaneously sarcopenic with elevated AT content. This BC profile had the worst OS in Caan et al.’s (2017) study, preceded in ascending order by sarcopenia alone, elevated AT alone, and with patients with “normal” weight surviving the longest [62]. Wang et al. (2022) confirmed this in a meta-analysis. Unlike adiposity alone, sarcopenic obesity predicted poorer OS, DFS, and postoperative complications in GI tract cancers [94]. In this review, of the 12 studies examining AT alone, seven found associations with poorer oncological or postoperative outcomes in complex RC [25,35,42,45,46,51,57]. Only three studies examined sarcopenic obesity; all of them found associations with postoperative complications [30,49] or OS and DFS [51].

#### 4.1.4. BC Change During Neoadjuvant Therapy

Only five of eleven studies comparing BC before and after nCRT found significant decreases in SM metrics [26,39,40,54,59]. It remains unclear to what degree of BC deterioration during nCRT is due to the treatment versus tumour biology. Xu et al. (2022) conducted a meta-analysis of preoperative SM change in gastrointestinal cancer patients undergoing neoadjuvant chemotherapy and/or radiotherapy. They concluded that neoadjuvant therapy was significantly associated with SM decrease and that loss was linked to reduced OS. However, only three RC studies were included, and no subgroup analysis was performed specifically for nCRT [94].

### 4.2. Potential Pitfalls

The papers included in this scoping review had a number of pitfalls ubiquitous to that which researchers and clinicians should be aware of both in terms drawing conclusions and in future study designs. These lie in the heterogeneous methodology, particularly in how BC is measured and how abnormal BC is defined.

#### 4.2.1. Total Lumbar Muscle vs. Psoas

Of the 33 studies utilising CT or MRI to quantify BC, 10 studies measured psoas rather than the total lumbar muscle within an axial slice [34,37,40,43,44,48,52,56,58,59]. The total lean body mass from DEXA is more highly correlated with the total lumbar muscle area compared to the single-slice psoas area (*r* = 0.94 vs. *r* = 0.74, respectively) [3]. In relation to the total lumbar SM surface area, psoas area measurements poorly-to-moderately correlate; poorly predict sarcopenia based on the literature cut-offs; and have reduced sensitivity for longitudinal change [95,96]. Furthermore, methodology exists in the literature where the psoas area is estimated using length and width measurements rather than direct segmentation [97]. Rutten et al. showed that such measurements have even lower correlations with total lumbar muscle area and high measurement errors; two studies in this scoping review used the psoas length/width method [48,52].

Such broad variations in anatomical region where BC is measured makes data synthesis challenging. Due to the aforementioned reasons, when a single-slice cross-sectional analysis of CT or MRI for BC is performed, the L3 total muscle area should be the preferred method rather than the psoas muscle alone. It is our opinion that clinicians should be cautious about drawing conclusions from articles using the psoas muscle alone.

#### 4.2.2. Segmentation Software

Segmentation is the process of labelling each pixel within a digital image as belonging to a specific category, with SM, SAT, and VAT in the context of BC. This is currently accepted as the de facto method for BC analysis from CT or MRI [98]. At least 14 different segmentation software programmes were identified in this review. Rollins et al. (2019) compared *Slice-O-matic* with *OsiriX* using L3-CT slices and observed significant differences in SM measurements [99]. Different segmentation software tools underestimate or overestimate SM and AT. The most commonly used SM-cut-offs in this review were from Prado et al. (2008), Martin et al. (2013), and Caan et al. (2017); all of these were derived with *Slice-O*-Matic [60,61,62]. Of the 13 studies that applied these, only two used *Slice-O*-Matic [35,57].

Researchers reviewing the literature or conducting original research should be aware of measurement variations produced by different software programmes. They should be especially wary of these differences when applying cut-off values for SM or AT. Efforts should be made to use the same software utilised by the seminal study producing the cut-offs. If different software is used, an equivalence study should be performed to show that the two software programmes produce similar SM and AT measurements.

#### 4.2.3. BC Cut-Off Values and Ethnicity

In addition to software choice, further caution is needed when applying cut-off values from another study. Twelve different cut-offs were identified for SM [3,60,61,62,63,64,68,100,101] and AT [65,66,67] measures across 18 studies. Of note, Fuijoka et al.’s (1987) VAT:SAT ratio >0.4 cut-off, used in three studies, is interestingly not sex-specific [64]; Clark et al. (2013) showed that the males in the cohort had significantly elevated ratios compared to females [25]. With exception of two cut-offs for SM percentage change [3,68], the remainder was sex ± BMI adjusted. However, ethnicity was not considered in the majority of these cut-offs. Jeng et al. (2018) showed significant variations in BC across White, Black, Asian, and Hispanic populations [102]. Choi et al. (2017), Park et al. (2018), and Abe et al. (2024) used South Korean/Japanese cut-offs [63,66,100], matching their East Asian geography. The remaining studies using other cut-off values were conducted in North America, West Europe, Turkey, India, and Australia. Whilst ethnicity was not outlined in any of these studies, these regions are considerably less homogenous in ethnic make-up than South Korea and Japan [103] and thus questions the appropriateness of applying external cut-offs. Authors should justify using external values for their cohort. Furthermore, the parameters for BC quantification used in the seminal study should be replicated. Two studies measured only the psoas area [48,52] and yet applied cut-off values based on the total L3 muscle area [60,104].

#### 4.2.4. Retrospective Methodology

Most of the papers included (31/35) were retrospective observational studies. This is reflective of the majority of published papers examining body composition in cancer patients. This carries the risk of selection bias inherent to such a study design, limits the strength of the conclusions drawn, and allows only for the observation of associations between variables rather than causal links. There is a need for more truly prospective cohort studies in the future, as well as interventional studies.

### 4.3. Knowledge Gaps and Areas for Future Research

#### 4.3.1. Opportunities for Meta-Analysis

Ten studies examined the relationship between BC and responses to neoadjuvant treatment, with six showing a significant association with sarcopenia. Whilst a formal assessment of study quality was beyond the scope of this review, there is potential for a meta-analysis of sarcopenia as a predictor of neoadjuvant therapy response in complex RC. A personalised prediction model of the success or futility of neoadjuvant therapy could empower patient-driven decision making and potentially avoid unnecessary morbidity.

#### 4.3.2. Body Composition and Beyond-TME Resectional Outcomes

Only one study focused on pelvic exenteration surgery. Interestingly, however, Van Rees et al. (2021b) found no association between sarcopenia and postoperative complications, nor with survival after pelvic exenteration [41]. Bedrikovetski et al. (2023) found similar results in pelvic exenteration for RC, gynaecological, and sarcoma combined [105]. Further studies in this group would be of value.

#### 4.3.3. Body Composition and Immunotherapy

Mismatch repair-deficient or microsatellite instability-high LARC has been shown to be highly responsive to anti-PD-1 immunotherapy, as seen in Hu et al.’s (2022) and Cercek et al.’s (2022) seminal work [15,16]. There will be a need to examine whether BC can predict toxicity as sarcopenia does for chemotherapy [77], as well as responses to treatment. Within colorectal cancer, at present, there are no studies examining anti-PD-1 immunotherapy from a BC lens to our knowledge. However, Dang et al. (2024) did very recently demonstrate an association between sarcopenia and VAT depletion with reduced progression-free survival in patients with metastatic colorectal cancer receiving cetuximab [106]. Other than colorectal cancer, another recent meta-analysis of BC in anti-PD-1 therapy for melanoma found that sarcopenia and elevated SAT radiodensity were associated with reduced overall and progression-free survival but not toxicity [107]. Due to its dramatic impact on previously prognostically poor disease, it is imperative that such work is replicated within complex RC.

#### 4.3.4. Automated 3D BC Analysis and Longitudinal Changes During Neoadjuvant Therapy

Automated solutions for single-slice segmentation are now widely available [108]. This allows for larger sample sizes to be analysed and makes the measurement of total BC surface area at the L3 level more accessible, negating the need to use the less reliable psoas area. Artificial intelligence also enables a 3D BC analysis of full multi-slice CT and MRI scans [97]. Software such as the *Data Analysis Facilitation Suite* by *Voronoi* automatically quantifies muscle, bone, and adipose, as well as solid and hollow organ volumes from whole CT scans covering any regions of anatomy [109]. For complex RC patients, this may reveal new prognostic biomarkers from the entire thoraco-abdominopelvic cavity beyond L3. Furthermore, since a larger region of tissue is captured, 3D analysis potentially provides a more accurate representation of whole BC. Pu et al. (2022) demonstrated volumetric thoracic BC was more accurate in predicting whole body SM, SAT, VAT, and bone compared to L3 [110]. Further evidence shows 3D multi-slice BC measures to be more precise and accurate at predicting longitudinal change [89]. Overall, such technology may provide a richer and more accurate BC profile of an individual patient, potentially making it more appropriate for targeted prehabilitation than a single L3 slice.

#### 4.3.5. Interventions for Abnormal BC

BC is a potentially modifiable determinant of outcomes. BC could be a valuable metric to be considered within complex RC multi-disciplinary team meetings. Opportunistic BC analysis from point-of-care imaging would allow for the convenient identification of abnormal BC phenotypes. In turn, these patients could undergo targeted prehabilitation. In non-cancer populations, there is evidence that sarcopenia and myosteatosis can be improved with exercise intervention [111,112], as well as with protein supplementation in the case of sarcopenia [113].

Yadav et al. (2024) demonstrated that muscle composition can be improved with multi-modal prehabilitation concurrently undertaken with nCRT and that this was associated with reduced postoperative complications after TME [59]. Moug et al. (2020) conducted a similar study; this was not included, as the patients were not exclusively LARC or LRRC. This was a sub-analysis of a feasibility trial, where patients having nCRT for RC were randomised to a control group and a prehabilitation group where they undertook a guided walking programme. There was a significantly greater increase in psoas muscle quantity in the intervention group [114].

Further interventional studies with larger sample sizes are warranted with longer follow up to determine whether BC improvements equate to oncological outcomes. Future studies should also aim to determine what the most effective interventions are for treating sarcopenia and myosteatosis. A 2023 network meta-analysis compared various combinations of aerobic exercise, resistance training, nutritional interventions, and electrical stimulation in terms of treating sarcopenia in non-cancer populations. The authors concluded that resistance training was the most effective intervention for improving muscle quantity, strength, and function [112].

Ultimately, there is a need for a prospective, international, multi-centre, and multi-modal interventional study examining BC in complex RC with regard to mortality, recurrence, postoperative complications, and neoadjuvant outcomes. Automated 3D analysis should be utilised, as well as capturing both cross-sectional and longitudinal BC. Due to the heterogeneity in methodology rife in the literature highlighted by this scoping review, such an endeavour would require a robust Delphi method with international representation for planning.

## 5. Conclusions

Complex RC has neoadjuvant and surgical treatment options, making it distinct within colorectal cancer. Similarly to non-complex colorectal cancer, this scoping review shows trends in the literature suggesting that sarcopenia predicts overall and disease-free survival in complex RC. Additionally, a novel meta-analysis of whether BC predicts responses to and tolerance of neoadjuvant therapy is now feasible. Research opportunities include further studies on pelvic exenteration outcomes; the harnessing of artificial intelligence-driven 3D analysis; assessing BC in the context of immunotherapy; and more emphasis of interventional studies on the treatment of abnormal BC. Pitfalls in BC research include using psoas alone as a surrogate measure for muscularity due to its lower correlation with total muscle quantity and increased measurement errors. Furthermore, caution should be exercised when using external cut-off values that have been applied to a study population to ensure appropriate application.

## Figures and Tables

**Table 1:** Summary of study characteristics.

Study Characteristic	Value
**Geographical region of origin** *[n (%)]*	
East Asia	, 13 (37.1%)
Europe	, 11 (34.1%)
Australia	, 5 (14.3%)
North America	, 3 (8.6%)
West Asia	, 2 (5.7%)
South Asia	, 1 (2.9%)
**Study aims** *[n (%)]*	
Investigate relationship between BC and LARC outcomes	, 31 (88.6%)
Generate BC based prediction models for LARC outcomes	, 3 (8.6%)
Investigate intervention to improve BC	, 1 (2.9%)
Investigate effect of nCRT on BC	, 8 (22.9%)
Investigate methodology for BC measurement	, 1 (2.9%)
**Study design** *[n (%)]*	
Retrospective cohort study of prospective database	, 5 (14.3%)
Retrospective cohort study	, 26 (74.3%
Prospective cohort study	, 2 (5.7%)
Prospective non-randomised experimental study	, 2 (5.7%)
**Disease characteristics of included patients** *[n (%)]*	
LARC	, 35 (100%)
LRRC	, 2 (5.7%)
Metastatic disease	, 5 (14.3%)
**Neoadjuvant therapy** *[n (%)]*	
NCRT	, 35 (100%)
Total neoadjuvant therapy	, 2 (5.7)
**Operative therapy** *[n (%)]*	
TME	, 31 (88.6%)
Beyond TME or pelvic exenteration	, 4 (11.4%)
Not applicable or unknown	, 4 (11.4%)
**Outcomes measured** *[n (%)]*	
Overall survival	, 16 (45.7%)
Disease-free survival	, 18 (51.4%)
Postoperative complications	, 17 (48.6%)
BC-related outcome	, 11 (31.4%)
Response to neoadjuvant or adjuvant treatment	, 10 (28.6%)
Complications to neoadjuvant or adjuvant treatment	, 6 (17.1%)
**Sarcopenia predicts response to nCRT** *[n (%)]*	
Yes	, 7 (20.0%)
No	, 1 (2.9%)
Not applicable	, 27 (77.1%)

**Table 2:** Summary of body composition analysis across studies.

**Source of BC analysis**	
CT source of BC	, 32 (91.4%)
MRI source of BC	, 3 (8.6%)
BIA source of BC	, 2 (5.7%)
**Region of BC measurement**	
L3 level	, 27 (77.1%)
Other lumbar level	, 3 (8.6%)
Umbilicus	, 2 (5.7%)
Mesorectum	, 1 (2.9%)
Thigh	, 1 (2.9%)
Whole body (BIA)	, 2 (5.7%)
**Body composition variables analysed**	
Skeletal muscle	, 32 (91.4%)
Psoas muscle	, 11 (31.4%)
Adipose tissue	, 12 (34.3%)
Tissue radiodensity	, 6 (17.1%)
Lean body mass	, 2 (5.4%)
**Method of BC measurement from CT/MRI**	
Manual or semi-automated segmentation	, 30 (85.7%)
Automated segmentation	, 1 (2.9%)
Diameter/width estimation of psoas	, 2 (5.7%)
Not applicable	, 2 (5.7%)
**Longitudinal BC analysis**	15 (42.9%)
**Timing of BC analysis**	
Pre neoadjuvant	, 33 (94.3%)
Post neoadjuvant	, 21 (60.0%)
**Method of categorisation of abnormal body composition**	
Cut-off from other studies	, 18 (51.4%)
Quartile or tertile or median or z scoring	, 12 (34.3%)
Bespoke optimal cut-off	, 2 (5.4%)
Continuous measures	, 7 (20.0%)

**Table 3:** Summary of study outcomes and findings. *BC—body composition; AT—adipose tissue*.

Outcome	Studies Examining Outcome (*n*, %)	Studies Finding Association Between BC and Outcome (*n*, %)	BC Parameter Linked to Outcome		
			Sarcopenia (*n*)	Myosteatosis (*n*)	AT Quantity (*n*)
Overall survival	16 (45.7%)	10 (62.5%)	8	2	0
Disease-free survival	18 (51.4%)	15 (83.3%)	11	0	4
Postoperative complications	17 (48.6%)	10 (58.8%)	7	1	3
Neoadjuvant treatment response	10 (28.6%)	9 (90.0%)	6	0	3
Neoadjuvant/adjuvant treatment tolerance	6 (17.1%)	2 (33.3%)	2	0	0

## Supplementary Materials

The following supporting information can be downloaded at: https://www.mdpi.com/article/10.3390/cancers17050846/s1, Table S1: Detailed summary of study characteristics.
