# Supplementary material for: A Scoping Review of the Implications and Applications of Body Composition Assessment in Locally Advanced and Locally Recurrent Rectal Cancer
Source: Cancers (Basel). 2025 Feb 28;17(5):846. doi: 10.3390/cancers17050846 (PMC11899338; doi:10.3390/cancers17050846)
Supplement: Supplementary file 1 [file cancers-17-00846-s001.zip › cancers-3415417-Supplementary.pdf]

Supplementary Table S1: Detailed summary of study characteristics

Study ID	Origin	Aims	Design	Setting	*n*	Inclusion criteria	LARC definition	M1 disease inclusion	Source of BC	Anatomical level of BC measures	If CT or MRI technique of BC tissue measurements	BC parameters measured	Software used	BC measures	Timing of BC analysis	Method of BC cut-offs	Surgical treatment	Outcomes measured	Findings
Clark 2013	United States	Assess links between BC & LARC outcomes	Retro-spective cohort study	Single centre	*99*	LARC with nCRT and resection	T3/4 and/ or >N0	No	CT	Between L4-L5	Manual/semi-automated segmentation	SAT VAT Peri-nephric AT	Siemans CT Leonardo	Cross-sectional	Pre-nCRT	Literature cut-off	TME	Postoperative complications OS & DFS Response to nCRT	Visceral obesity associated with reduced DFS. Visceral obesity associated with reduced rate of post nCRT downstaging
West 2014	UK	Effect of nCRT on BC	Experi-mental study	Single centre	*12*	LARC with nCRT	T3/4+CRM <1mm and/or >N0	No	MRI	Quadricep	Phosphorus MR spectroscopy	SM mitochondrial function	N/A	Longitu-dinal	Pre-nCRT Post-nCRT	Left as continuous variable	N/A	VO2 at peak exercise and at AT Phosphocreatine recovery rate.	Skeletal muscle mitochondrial function decreases after nCRT
Levolger 2017	The Nether-lands	Assess links between BC & LARC outcomes Effect of nCRT on BC	Retro-spective cohort study; prospective database	Single centre	*122*	LARC with nCRT and resection	T3/4+CRM <1mm <1mm and/or >N0	No	CT	L3	Manual/semi-automated segmentation	Skeletal muscle Longitudinal change	FatSeg	Longitu-dinal	Pre-nCRT Post-nCRT	Tertiles. Literature cut-off	TME Beyond-TME Pelvic exenteration	OS & DFS	Sarcopenia predicted reduced DFS
Choi 2017	South Korea	Assess links between BC & LARC outcomes	Retro-spective cohort study	Single centre	*188*	LARC with nCRT and resection	T3/4 and/ or >N0	No	CT	L3	Manual/semi-automated segmentation	SM SAT VAT	Advantage Windows Workstation 4.6	Cross-sectional	Pre-nCRT	Literature cut-off	TME	Postoperative complications OS & DFS	Sarcopenia predicted OS. Visceral obesity predicted DFS on univariate analysis. BC did not predict postoperative outcomes
Takeda 2018	Japan	Assess links between BC & LARC outcomes	Retro-spective cohort study	Single centre	*144*	LARC with nCRT and resection	T3/4 and/ or >N0	No	CT	L3	Manual/semi-automated segmentation	SM	Synapse3D	Cross-sectional	Pre-nCRT	Quartile	TME	Postoperative complications; OS & DFS	Sarcopenia predicted reduced OS. Sarcopenia predicted reduced DFS
Berkel 2018	The Nether-lands	Assess links between BC & LARC outcomes	Retro-spective cohort study	Single centre	*99*	LARC with nCRT and resection	T3/4 and/ or >N0 and/or CRM <1mm	No	CT	L3	Manual/semi-automated segmentation	SM Psoas muscle SM radio-density	TeraRecon	Cross-sectional	Pre-nCRT	Median	TME	Postoperative complications OS	Reduced SM radiodensity associated with overall and major complications. Sarcopenic obesity associated with overall complications
Park 2018	South Korea	Assess links between BC & LARC outcomes	Retro-spective cohort study	Single centre	*65*	≥65 years old LARC neoadj/ adjuvant CRT and resection	T3/4 and/ or >N0	No	CT	L3	Manual/semi-automated segmentation	SM	Unspecified	Cross-sectional	Pre-nCRT Post-nCRT	Literature cut-off	TME	OS & DFS	Sarcopenia predicted reduced OS
Chung 2019	South Korea	Assess links between BC & LARC outcomes Effect of nCRT on BC	Retro-spective cohort study	Single centre	*93*	LARC with nCRT and resection	Not defined	No	CT	L3	Manual/semi-automated segmentation	SM SAT VAT	TeraRecon	Longitu-dinal	Pre-nCRT Post-nCRT	Left as continuous variable	TME	Postoperative complications OS & DFS Predictors of severe SM loss	T4 predicted severe SMI loss. Sarcopenia predicted reduced OS. SMI decrease post nCRT predicted reduced OS
Jochum 2019	United States	Assess links between BC & LARC outcomes	Retro-spective cohort study	Single centre	*47*	LARC with nCRT and resection	T3/4 and/ or >N0	No	CT	L3	Manual/semi-automated segmentation	SM	Mimics	Cross-sectional	Pre-nCRT Post-nCRT	Literature cut-off	TME	Postoperative complications	Sarcopenia associated with overall postoperative complications
Fukuoka 2019	Japan	Assess links between BC & LARC outcomes	Retro-spective cohort study	Single centre	*47*	LARC having either nCRT or	T3/4 and/ or >N0	No	CT	Umbilicus	Manual/semi-automated segmentation	Psoas muscle Longitudinal change	Unspecified	Longitu-dinal	Pre-nCRT Post-nCRT	Bespoke optimal cut-off	TME	Postoperative complications DFS	>10% psoas muscle loss associated with reduced DFS

		Effect of nCRT on BC				chemo alone.													
de Nardi 2019	Italy	Assess links between BC & LARC outcomes	Retro-spective cohort study; prospective database	Single centre	*52*	LARC with nCRT and resection	T3/4 and/ or >N0	No	CT	L3	Manual/semi-automated segmentation	SM SAT VAT Longitudinal change	Slice-O-matic	Cross-sectional	Pre-nCRT Post-nCRT	Literature cut-off	TME	Postoperative complications OS & DFS	SMA loss associated with severe complications. SM loss and SAT loss associated with reduced DFS on univariate analysis. Multivariate analysis not performed due to small sample size. A further subgroup analysis on N0 patients showed SM and SAT loss still predicted DFS on univariate analysis.
Yama-no 2020	Japan	Assess links between BC & LARC outcomes Effect of nCRT on BC	Prospective cohort study	Multi centre	*41*	LARC with nCRT and planned for surgery.	T3/4 and/ or >N0-2	No	BIA	BIA	111	Lean body mass	BIA	Longitu-dinal	Pre-nCRT Post-nCRT	Left as continuous variable	N/A	nCRT completion NCRT-related toxicity Disease-free survival	No meaningful associations with LBM. However, there was missing data in 50% of cases
Olmez 2020	Turkey	Assess links between BC & LARC outcomes	Retro-spective cohort study	Single centre	*61*	LARC with nCRT and resection	Not defined	No	CT	L3	Manual/semi-automated segmentation	SM Psoas muscle	Unspecified	Cross-sectional	Pre-nCRT	Literature cut-off	TME	Postoperative complications; Response to nCRT	Sarcopenia predicted reduced complete response to nCRT
van Rees 2021a	The Nether-lands	Assess links between BC & LARC outcomes	Retro-spective cohort study	Single centre	*746*	LARC and LRRC with capecita-bine-containin g nCRT.	Not defined	Yes	CT	L3	Manual/semi-automated segmentation	SM SM radio-desity	FatSeg	Cross-sectional	Pre-nCRT	Literature cut-off	N/A	NCRT-related toxicity	BC did not predict toxicity to CRT
Hel-dens 2021	The Nether-lands	Effect of nCRT on BC	Retro-spective cohort study	Single centre	*25*	LARC with nCRT	T3/4+CR M <1mm and/or >N0	Yes	CT	L3	Manual/semi-automated segmentation	SM Change in SMI	Slice-O-matic	Longitu-dinal	Pre-nCRT Post-nCRT	Literature cut-off	TME	Body composition changes Functional capacity changes	Steep ramp test performance and SMI declined pre- and post CRT. However, 9 of 25 patients could not have CT analysis. BC analysis only performed on 16 patients
Abe 2021a	Japan	BC-based prediction model for LARC outcomes	Retro-spective cohort study	Single centre	*225*	LARC with nCRT and resection	T3/4 and/ or >N0	No	CT	L3	Manual/semi-automated segmentation	Psoas muscle	Ziosoft	Longitu-dinal	Pre-nCRT Post-nCRT	Quartile	TME	OS & DFS; predictors of malnutrition and sarcopenia.	Sarcopenia predicts OS
van Rees 2021b	The Nether-lands	Assess links between BC & LARC outcomes Effect of nCRT on BC	Retro-spective cohort study	Single centre	*227*	Patients with LARC undergoin g pelvic exenterati ons.	T4+CRM <1mm and/or N2 disease	Yes	CT	L3	Manual/semi-automated segmentation	SM SM radio-desity Longitu-dinal change	FatSeg	Longitu-dinal	Pre-nCRT Post-nCRT	Literature cut-off Quartile	Pelvic exentera-tion	Postoperative complications; OS; Primary outcome was postoperative complications	MUST score 2 or higher independently predicted major complications; SM-metrics did not. MUST did not predict OS. Myosteatosis (low SM radiodensity) did on univariate (0.050), but not multivariate analysis.
Dilek 2021	Turkey	Assess links between BC & LARC outcomes	Retro-spective cohort study	Single centre	*88*	LARC with nCRT and resection	Not defined	No	CT	Mesorect um	Manual/semi-automated segmentation	SM SAT VAT Mesorectal fat	Intelli SpacePhillip s	Cross-sectional	Pre-nCRT	Left as continuous variable	TME	Response to nCRT Relationship between mesorectal fat and BC	Mean mesorectal fat higher in groups with response to nCRT
Horie 2021	Japan	Assess links between BC & LARC outcomes	Retro-spective cohort study	Single centre	*46*	LARC with nCRT and resection	T3/4 and/ or >N0	No	CT	L3 Total Iliopsoas volume	Manual/semi-automated segmentation	Psoas muscle	Ziosoft	Longitu-dinal	Pre-nCRT Post-nCRT	Medians	TME	Postoperative complications; OS & DFS	Sarcopenia predicted reduced DFS
Abe 2021b	Japan	Assess links between BC & LARC outcomes	Retro-spective cohort study	Single centre	*234*	LARC with nCRT and resection	T3/4 and/ or >N0	No	CT	L3	Manual/semi-automated segmentation	Psoas muscle	Ziosoft	Cross-sectional	Pre-nCRT Post-nCRT	Quartile	TME	OS & DFS; Clinicopathologic al predictors of sarcopenia	Sarcopenia predicts OS. Sarcopenia predicts DFS

Liu 2022	China	Assess links between BC & LARC outcomes	Retro-spective cohort study of prospec-tive data	Single centre	*122*	LARC with nCRT and resection	Not defined	No	CT	L3	Manual/semi-automated segmentation	SM SAT VAT SM radio-desity	ImageJ	Longitu-dinal	Pre-nCRT Post-nCRT	Quartile	TME	Postoperative complications; OS & DFS; Response to nCRT	Low SM radiodensity and total AT loss associated with ileus. Sarcopenia predicted reduced DFS and CSS. Myosteatosis predicted reduced CSS. Sarcopenia predicted response to nCRT.
Bocca 2022	UK	Assess links between BC & LARC outcomes	Retro-spective cohort study; prospective data	Single centre	*78*	LARC treat with nCRT/ TNT, and resection	T3/4 and/ or >N0 and/or CRM <1mm and/or EMVI	No	CT	L3	Manual/semi-automated segmentation	SM SAT VAT	CoreSlicer.com	Cross-sectional	Pre-nCRT Post-nCRT	Literature cut-off	TME	Postoperative complications; Length of stay	Visceral obesity associated with increased LOS. Elevated V/S associated with increased LOS. Sarcopenic obesity associated with increased LOS.
Melucci 2022	United States	Evaluation of BC measurement technique	Retro-spective cohort study	Single centre	*16*	LARC with available BIA and hand-grip strength values	Not defined	No	CT MRI BIA	L3 for CT L5 for MRI	Manual/semi-automated segmentation	SM LBM	Slice-O-Matic	Cross-sectional	Pre-nCRT	Left as continuous variable	N/A	Agreement between the above measures	Strong correlation between CT and MRI estimations of LBM. Stronger correlations between BIA-LBM and MRI-LBM than BIA vs CT. CT LBM - higher correlation with HGS than MRI. Note this study only measures correlation and not agreement.
Bedriko-vetski 2023a	Austra-lia	Assess links between BC & LARC outcomes	Prospective cohort study	Multi centre	*118*	LARC with TNT and resection	Not defined	Yes	CT	L3	Diameter and width estimation	Psoas muscle	None	Cross-sectional	Pre-nCRT Post-nCRT	Literature cut-off	TME Pelvic exentera-tion	Response to nCRT; NCRT-related toxicity; Primary outcome of overall complete response. Toxicity was a secondary outcome	Sarcopenia predicted reduced complete response to nCRT
Mallet-Boutboul 2023	France	Assess links between BC & LARC outcomes	Retro-spective cohort study	Single centre	*100*	LARC with nCRT and resection	T3/4 and/ or >N0	No	CT	L3	Manual/semi-automated segmentation	SM SAT VAT	Synapse3D	Longitu-dinal	Pre-nCRT Post-nCRT	Literature cut-off	TME	Postoperative complications OS & DFS Response to nCRT	Sarcopenia - reduced DFS. Sarcopenia & viscerally obese groups - higher LOS. Sarcopenia - higher 90-day morbidity. Visceral obese groups - higher rate of unplanned readmission.
Gartrell 2023	Austra-lia	Assess links between BC & LARC outcomes	Retro-spective cohort study	Single centre	*132*	LARC with nCRT and resection	T3/4 and/ or >N0	No	CT	L3	Manual/semi-automated segmentation	SM	Slice-O-matic	Cross-sectional	Pre-nCRT	Tertiles	TME	OS & DFS	Sarcopenia predicted reduced OS
Tsch-ann 2023	Austria	Assess links between BC & LARC outcomes	Retro-spective cohort study	Single centre	*96*	LARC with nCRT and resection	T3/4 and/ or >N0	No	CT	Umbilicus	Manual/semi-automated segmentation	SM SAT VAT Visceral-to-total-fat ratio Subcutaneous-to-total-fat ratio	Horos	Cross-sectional	Post-nCRT	Z-scoring Literature cut-off	TME	Postoperative complications OS & DFS Early postop complications was primary	Higher BMI patients were likelier to have excess SAT and VAT; no difference in muscle. Univariable - overall morbidity predicted by sarcopenia and sarcopenic obesity. Univariable - Sarcopenic obesity predicts OS and DFS.
Bedriko-vetski 2023b	Austra-lia	Assess links between BC & LARC outcomes	Retro-spective cohort study; prospective data	Multi centre	*714*	LARC with nCRT	Not defined	Yes	CT	L3	Diameter and width estimation	Psoas muscle	None	Cross-sectional	Pre-nCRT	Literature cut-off	TME Pelvic exentera-tion	Response to nCRT	Sarcopenia was not associated with tumour regression. Sarcopenia not associated with complete response to nCRT.
Yang 2024	China	BC-based prediction model for LARC outcomes	Retro-spective cohort study	Single centre	*214*	LARC with nCRT	T3/4 and/ or >N0	No	CT	L3	Manual/semi-automated segmentation	SM	MIM software	Cross-sectional	Pre-nCRT	Left as continuous variable	N/A	NCRT-related toxicity	The radiomics score was the only independent predictor of Grade 3/4 GI toxicity. However, the final model also included the SMI. 9 radiomic features and SMI were included in the final model. All predictive performance on training, and

																			test data were improved by adding radiomics to the SMIT alone (AUC >0.8 for training and internal; 0.75 for external).
Yang 2023	China	BC-based prediction model for LARC outcomes	Retro-spective cohort study	Single centre	*291*	LARC with nCRT and resection	T3/4 and/ or >N0	No	CT	L3	Manual/semi-automated segmentation	SM SAT VAT	Slice-O-Matic	Longitu-dinal	Pre-nCRT Post-nCRT	Quartile	TME	Response to nCRT; Changes in BC	SMI decreased significantly after nCRT. Sarcopenia predicted reduced complete response.
Wei 2024	Austra-lia	Assess links between BC & LARC outcomes	Retro-spective cohort study	Single centre	*214*	LARC with nCRT and resection	Not defined	No	CT	L3	Automated segmentation	SM SAT VAT SM, SAT, VAT radiodensity	In-house AI algorithm	Cross-sectional	Pre-nCRT	Quartile	TME	Response to nCRT;	SM volume predicted complete response to nCRT.
Yassaie 2024	Austra-lia	Assess links between BC & LARC outcomes Effect of nCRT on BC	Retro-spective cohort study	Single centre	*92*	LARC with nCRT	Not defined	No	CT	L4	Manual/semi-automated segmentation	Psoas muscle Longitu-dinal change	Unspecified	Longitu-dinal	Pre-nCRT Post-nCRT	Left as continuous variable	TME	Anastomotic leak	Sarcopenia predicted reduced rate of complete response to nCRT
Chiloiro 2024	Italy	Assess links between BC & LARC outcomes	Retro-spective cohort study	Single centre	*628*	LARC with nCRT and resection	T2-4 and/ or n0-1	No	CT	L3	Manual/semi-automated segmentation	SM SAT VAT Intermus-cular AT SM radio-desity	Slice-O-matic	Cross-sectional	Pre-nCRT	Literature cut-off	TME	OS; DFS; Response to nCRT; NCRT-related toxicity; Primary was interruption of CRT	Sarcopenia predicted radiotherapy interruption. Reduced SM radiodensity predicted reduced OS. Elevated VAT predicted DFS. Elevated SM predicted better response to nCRT.
Abe 2024	Japan	Assess links between BC & LARC outcomes	Retro-spective cohort study	Single centre	*93*	LARC with nCRT, resection, and adjuvant chemothe rapy.	T3/4 and/ or >N0	No	CT	L3	Manual/semi-automated segmentation	Psoas muscle	Ziosoft	Cross-sectional	Post-nCRT	Literature cut-off	TME	DFS; Adjuvant chemotherapy dose limiting toxicity Adjuvant chemotherapy-related toxicity >Grade II	Sarcopenia was not associated with dose-limiting toxicity.
Yadav 2024	India	Evaluate intervention for BC.	Experi-mental study	Single centre	100	LARC with nCRT and planned for surgery.	Not defined	No	MRI	L4	Manual/semi-automated segmentation	Psoas muscle	Unspecified	Longitu-dinal	Pre-nCRT Post-nCRT	Literature cut-off	TME	Postoperative complications: Primary outcome was psoas muscle index.	Prehabilitation results in a significant reduction in sarcopenia frequency post-nCRT, compared to control.
